# A Novel Patient Values Tab for the Electronic Health Record: A User-Centered Design Approach

**DOI:** 10.2196/21615

**Published:** 2021-02-17

**Authors:** Anjali Varma Desai, Chelsea L Michael, Gilad J Kuperman, Gregory Jordan, Haley Mittelstaedt, Andrew S Epstein, MaryAnn Connor, Rika Paula B Villar, Camila Bernal, Dana Kramer, Mary Elizabeth Davis, Yuxiao Chen, Catherine Malisse, Gigi Markose, Judith E Nelson

**Affiliations:** 1 Memorial Sloan Kettering Cancer Center New York, NY United States; 2 Department of Medicine Weill Cornell Medical College New York, NY United States

**Keywords:** electronic health record, health informatics, supportive care, palliative care, oncology

## Abstract

**Background:**

The COVID-19 pandemic has shined a harsh light on a critical deficiency in our health care system: our inability to access important information about patients’ values, goals, and preferences in the electronic health record (EHR). At Memorial Sloan Kettering Cancer Center (MSK), we have integrated and systematized health-related values discussions led by oncology nurses for newly diagnosed cancer patients as part of routine comprehensive cancer care. Such conversations include not only the patient’s wishes for care at the end of life but also more holistic personal values, including sources of strength, concerns, hopes, and their definition of an acceptable quality of life. In addition, health care providers use a structured template to document their discussions of patient goals of care.

**Objective:**

To provide ready access to key information about the patient as a person with individual values, goals, and preferences, we undertook the creation of the Patient Values Tab in our center’s EHR to display this information in a single, central location. Here, we describe the interprofessional, interdisciplinary, iterative process and user-centered design methodology that we applied to build this novel functionality as well as our initial implementation experience and plans for evaluation.

**Methods:**

We first convened a working group of experts from multiple departments, including medical oncology, health informatics, information systems, nursing informatics, nursing education, and supportive care, and a user experience designer. We conducted in-depth, semistructured, audiorecorded interviews of over 100 key stakeholders. The working group sought consensus on the tab’s main content, homing in on high-priority areas identified by the stakeholders. The core content was mapped to various EHR data sources. We established a set of high-level design principles to guide our process. Our user experience designer then created wireframes of the tab design. The designer conducted usability testing with physicians, nurses, and other health professionals. Data validation testing was conducted.

**Results:**

We have already deployed the Patient Values Tab to a pilot sample of users in the MSK Gastrointestinal Medical Oncology Service, including physicians, advanced practice providers, nurses, and administrative staff. We have early evidence of the positive impact of this EHR innovation. Audit logs show increasing use. Many of the initial user comments have been enthusiastically positive, while others have provided constructive suggestions for additional tab refinements with respect to format and content.

**Conclusions:**

It is our challenge and obligation to enrich the EHR with information about the patient as a person. Realization of this capability is a pressing public health need requiring the collaboration of technological experts with a broad range of clinical leaders, users, patients, and families to achieve solutions that are both principled and practical. Our new Patient Values Tab represents a step forward in this important direction.

## Introduction

### Background

Besides inadequate personal protective equipment and limited intensive care surge capacity, the COVID-19 pandemic has shined a harsh light on another critical deficiency in our system of health care delivery: our inability to access important information about patients’ values, goals, and preferences in the electronic health record (EHR) [1-3]. All over the United States, frontline clinicians in the field, emergency departments, intensive care units (ICUs), and on rapid response teams and hospital floors have struggled with urgent decisions about the use of life-supporting technologies to treat serious complications of COVID-19 infection without sufficient information about the patient as a person, what means most to this individual, how the patient defines living well, and whom the patient trusts to make important decisions about health care [4,5]. Such information is rarely accessible even for older adults and others with underlying diseases, such as cancer or chronic comorbid conditions, who are most vulnerable. Either this information was not previously elicited or, although it was discussed, it was not documented or is difficult to find in the EHR [6-9].

This is not a new problem, and it will certainly persist after COVID-19 in the absence of major innovative efforts. Although many clinicians consider the EHR to pose a barrier to patient-centered care by literally shifting the attention from the patient to the computer screen [10-12], the EHR is also a potentially powerful tool that can support clinician-patient communication, team collaboration, personalized and respectful care, continuity across settings, patient engagement, and shared decision making in accordance with patients’ individual needs and priorities [13,14]. Several recent initiatives have utilized digital platforms and tools (eg, documentation templates, automated prompts, and electronic order sets) to optimize documentation of advance care planning and goals of care discussions [15-17]. Some have focused primarily on the documentation of advance care planning in patients who are older or have advanced disease [18,19]. A more recent initiative leverages the Epic EHR by assembling information about serious illness conversations, including prognostic information given by clinicians to the patient and family, the patient’s understanding of the course of the illness, hopes and worries, priorities, and clinician recommendations, into a new EHR template [20].

At Memorial Sloan Kettering Cancer Center (MSK), we integrated and systematized health-related values discussions led by oncology nurses for newly diagnosed cancer patients as part of routine comprehensive cancer care, regardless of the patient’s stage, prognosis, or treatment intent [21,22]. These discussions are revisited quarterly or as deemed appropriate after prespecified clinical events (eg, progression of disease through first-line therapy, hospitalization, or admission to the ICU). In this model, communication encompasses not only the patient’s wishes for care at the end of life but also more basic and holistic personal values, including sources of strength, concerns, hopes, definition of an acceptable quality of life, and what the patient wants the clinical team to know about them as a person in order to provide the best care and preserve dignity [22]. In addition, oncologists and other physicians and advanced practice providers use a structured template to document their discussions of patient goals of care, which may address the expected course of the patient’s illness, intent of the current treatment, goals identified by the patient, preferences for end-of-life care, and, if relevant, hospice enrollment.

### The Patient Values Tab: Concept and Design

To provide ready access to these crucial communications between clinicians and patients as well as key information about the patient as a person with individual values, goals, and preferences, we undertook the creation of the Patient Values Tab in our center’s EHR to display this information in a single centralized location. Our institution’s EHR platform is Allscripts Sunrise Clinical Manager (Allscripts Healthcare LLC), which organizes data displays into tabs, as do many other EHR platforms. Here, we describe the interprofessional, interdisciplinary, iterative process and user-centered design methodology we applied to build this novel functionality [23].

## Methods

### Working Group and Stakeholder Interviews

We first convened a working group of experts from multiple departments, including medical oncology, health informatics, information systems, nursing informatics, nursing education, and supportive care (a multidisciplinary palliative care service), and a user experience designer. Members of the working group were selected for their previous experience leading large-scale institutional initiatives and unique expertise in the integration of supportive care and oncology. This core team met every 2 weeks to collaborate on the development and design of the new tab.

To understand the needs and perspectives of a broad range of institutional stakeholders, we also conducted in-depth, semistructured, audiorecorded interviews of over 100 key stakeholders based on a written guide prepared by the core team. Analysis of these interviews, which is reported in a separate publication (which includes selected stakeholder comments in an appendix), was used to inform content and format of the tab [24]. As active contributors to the creative process, those who were interviewed were more inclined to buy in to the ultimate product. In addition, many of these stakeholders held leadership positions within divisions and departments and went on to share enthusiasm about the upcoming tab with their colleagues, enhancing the visibility of the ongoing development effort among a broader group of users.

### Mapping Tab Content

The core team sought consensus on the Patient Values Tab’s main content, homing in on high-priority areas identified in the stakeholder interviews while also incorporating their input on format and considering suggestions on the logistics of the implementation process. The core content was mapped to various data sources within the EHR (as shown in Multimedia Appendix 1).

For example, oncology nurses use a structured document entitled “Assessment, Patient Personal Values” to summarize their values discussions. The patient’s preferred name, language, and communication preferences are elicited through the digital patient portal system via an electronic care questionnaire, which the nurse verifies and updates as needed at the first clinic visit on the nursing health assessment. Education about health care proxy (HCP) and other advance directives is provided in the hospital by patient representatives, who also record designation of and information about the HCP in a specific clinical document.

### High-Level Design Principles

As the data sources were clarified, we also established a set of high-level design principles (shown in Textbox 1) to guide this process.

**High-level design principles.**

1. The Patient Values Tab should provide users with an at-a-glance understanding of the patient as a person.
2. The Patient Values Tab should offer easy access to data that can be viewed in aggregate in the context of other relevant information to support clinical decision making.
3. The Patient Values Tab will specify the context in which the information was collected (with the source and date the information was updated).
4. The Patient Values Tab will be in a read-only format (ie, the data displayed will be captured and edited elsewhere).
5. The Patient Values Tab will contain the most high-yield information while presenting this information in a succinct, streamlined way to minimize cognitive load for users.
6. The Patient Values Tab will be accessible to all health care team members across the spectrum of patient care.

One such principle was that the tab would be in a read-only display format, populated with existing source documents in the EHR that could not be directly edited in the tab itself. We chose this approach because we and various key stakeholders we interviewed were concerned that the content would become unwieldy and unstable, ultimately leading to inefficiency for users if multiple modifications were allowed. We concluded that reliance on existing workflows and processes would reduce the cognitive load for clinicians and enable a communal responsibility for the underlying documentation, with important roles for various team members as contributors to a shared understanding of the patient as a person.

### User Testing

The next step was close collaboration with our user experience designer to create and refine wireframes of the tab design. During this process, the designer conducted usability testing with physicians, nurses, and other health professionals. All participants were asked to provide general usability feedback on the design. To obtain more detailed feedback, participants were presented with specific clinical tasks calling for the use of information contained in the Patient Values Tab in scenarios that the core team generated and adapted for particular roles and responsibilities. For example, in one scenario, the physician was preparing to “discuss serious results with the patient,” while the nurse needed to “provide information for medication management.” This process revealed a lack of familiarity among users with some of the underlying documentation that sourced the data displayed in the tab. To address this issue, we created a frequently asked questions tile (ie, section) in the tab including basic information about data sources and directing users to the appropriate underlying document to update the information, if needed.

### Refinement and Validation

Additional design refinements incorporated (1) suggestions from the user experience designer (eg, creating a banner at the top of the tab with the preferred name information, color coding the different categories of information to help users recognize and navigate among them quickly, including a separate feedback tile within the tab inviting users’ input and comments about their experience using the tab); (2) input from the core team (eg, placing the goals of care discussions and the nurse values summary in the most central, prominent positions in the tab; extracting and separately displaying the essential information regarding emergency contact, HCP, and next of kin for rapid access during clinical emergencies, with relevant advance directive scanned forms located below this information); and (3) insights gleaned from the stakeholder interviews (eg, providing expedited access to consultant notes from supportive care, psychiatry, and ethics as the highest yield and most consistently complete sources of information about patient values and personhood). During this iterative process, the designer shared mock-up options and elicited feedback from the core team via email and throughout the series of biweekly core team meetings.

Members of our core team with informatics expertise conducted data validation testing to ensure the fidelity of the information displayed in the tab to the underlying source of this information (ie, confirming that the tab displayed the correct information from the intended source document in the intended format for the correct patient). In addition, to detect delays in displaying information to the user that might decrease usability, we assessed the time to launch the tab content for selected patients. If time delays were identified, we worked with the information systems team to identify the source of the delay so that it could be rectified; specifically, technical optimizations were made to bring the load times in line with other feature load times in our EHR (ie, no longer than a few seconds).

## Results

### Initial Deployment and Implementation

We have already deployed the Patient Values Tab to a pilot sample of users in the Gastrointestinal (GI) Medical Oncology Service at our center, including the physicians, advanced practice providers (APPs), nurses, and administrative staff. We chose this service for this first phase of our pilot deployment because it is the largest solid tumor service at MSK, with approximately 40 physicians varying in age, gender, race and ethnicity, number of years in clinical practice, and disease focus who care for a large number of cancer patients with diverse demographics (eg, age, gender) and types and stages of cancer with wide variability in the pace of the disease process and overall clinical course. Patients of the GI Medical Oncology Service represent the largest proportion of admissions to our Memorial Hospital, thus frequently involving both outpatient and inpatient teams in their care.

We have executed a detailed implementation plan, which includes (1) discussion with the physician, APP, and nursing leaders of the service about the tab and its integration in clinical care by that service; (2) email notification to users about the availability and basic content of the new tab; and (3) presentations encompassing key features and functionality of the tab together with options for incorporating its use in regular clinical workflows. For each of these steps, we have targeted the professional groups individually, since they have different needs, roles, and workflows in providing patient care, although ultimately, they must all collaborate and communicate as a team to optimize this care.

### Further Iteration and Evaluation

Learnings from this pilot will inform further refinement of the Patient Values Tab before broader implementation. We are measuring usage of the tab through audit logs, which specify the time, user, and patient involved each time it is launched. In addition, we have included a feedback tile within the tab itself that asks in closed-ended items for users’ broad impressions (“It’s great,” “It’s okay,” or “It needs improvement”) and provides space for free-text comments. In-depth feedback will be gathered from selected clinicians through brief individual interviews. We plan to select these clinicians based on audit log data indicating those who are low adopters or high adopters in terms of the frequency and timing of their tab use. Through the interviews, we will explore whether the tab fits with the clinician’s workflow and patterns of use (or lack of use) in clinical practice, the perceived value of the tab to the clinician, and suggestions for improvement (eg, with respect to content and format).

Guided by this diverse user input, we have continued cycles of refinement and deployment to additional groups incrementally, with a plan for institution-wide rollout by the fall of 2020. The core team is continuing its biweekly meetings, monitoring every step in the implementation and evaluation process, and collaborating to address and incorporate user feedback in order to ensure that the use of the tab is maximized and sustained.

### Early Findings

We already have early evidence of the positive impact of this EHR innovation. Audit logs show increasing use among different user groups. Among users who have engaged with the feedback section within the tab, 25 of 48 (52%) users reported that “it is great” compared with 18 of 48 (38%) users who reported that “it needs improvement” and provided specific constructive suggestions and 5 of 48 (10%) users who reported that “it is okay.” Many of these initial user comments have been enthusiastically positive (Textbox 2).

**Illustrative quotes.**

“[I] love the easy access to [health care proxy] and Advance directives!”

“Being able to quickly check the patient's preferred name is incredibly helpful!”

“I like that it captures information from many different areas and placed in one area. I will be using this tab.”

“Numerous tiles you have developed give the patient a ‘voice’ in our [electronic health record]. Strong work….This will be a great advance in Cognitive Support for our clinical teams.”

Users also suggested specific enhancements to the tab’s content quality and design.

Our early pilot data have also revealed potential barriers to large-scale implementation, which include perceived lack of time to engage with the tab, competing priorities, incomplete or inaccurate tab content, technical glitches, and difficulty remembering to launch the tab as part of routine workflow. To address these challenges, we have enacted several proactive educational strategies when deploying to additional user groups, including (1) emphasizing that the tab is a display-only feature that is intended to save time by consolidating key information in a central location, (2) describing the processes by which incomplete or inaccurate tab content can be updated by the user, and (3) highlighting the “share feedback” section as a means for providing feedback on technical (or content-related) glitches that can be addressed by the Patient Values Tab interdisciplinary working group. In all circumstances, the Patient Values Tab working group follows up with each individual user who provides feedback to ensure closed-loop communication and enhance transparency in the ongoing effort to refine and optimize the tab’s functionality.

We are also increasingly receiving reports of specific patient situations in which the information displayed in the tab, including the outpatient oncology nurse’s values summary, allowed the hospital team to support families in difficult decisions about the use of intensive care therapies. In one such case, the family described hearing the ICU physician reading the values summary aloud as feeling that the patient, who no longer was conscious and had not discussed this type of situation with them previously, “was in the room, speaking directly to them,” clarifying his priorities, providing guidance, and relieving them of burden and guilt in deciding to limit life support at that time. Immediate access that the Patient Values Tab provides to primary physicians’ goals of care discussions greatly facilitates the work of our emergency department and rapid response team clinicians, who must act quickly in emergencies.

Similarly, we are hearing that the communication preferences section within the Patient Values Tab is particularly helpful to teams as they are preparing for family meetings and goals of care discussions. For example, one Patient Values Tab user recounted:

I did use it actually this week.…We were going to go have an impromptu family meeting and the attending physician asked, “Would the patient want to be involved?” and I said, “Well, let’s look at the Patient Values Tab”….We clicked there and it said “patient wants a lot of information with her partner present”…so…we used it in real time.

In this case, knowing how the patient preferred to receive medical information (ie, in detail) and with whom present (ie, her partner) helped guide the health care team’s approach to having a goals of care conversation at a timely moment.

## Discussion

Even at this pilot stage of deployment, it is becoming clear that the Patient Values Tab enables care and decision making that honors the personhood and values of our patients. During the COVID-19 pandemic, as life-threatening emergencies have occurred without warning to patients who were then too ill to speak for themselves, we have learned—again, in a more painful but perhaps more enduring lesson—that high-quality care is heavily dependent on immediate access to this crucial information. As implementation and usage of the tab expands and our center moves beyond the current COVID-19 crisis, we will be able to examine its impact on a variety of outcomes at the level of the patient, clinician, work process, and health care system. Although the Patient Values Tab was built in MSK’s specific EHR (ie, Allscripts), this software can be configured in other EHR platforms as well, including Epic (the predominant platform in the United States). Further research is needed on the best ways to optimize and enhance the patient centeredness of various EHR systems through the synergistic integration of tools that capture broader, holistic patient values (eg, the Patient Values Tab), with ongoing efforts that are primarily focused on advance care planning (eg, Epic’s Advance Care Planning tab).

All EHRs contain extensive quantitative information and voluminous data about patients that are mostly impersonal. Now, it is our challenge and obligation to enrich the EHR with information about the patient as a person, which is rarely included or readily accessible. Patient-centered care is highly prioritized by the Institute of Medicine [25], patients and families, and professional caregivers [26], but it can only be delivered if the patient’s personal values are as prominent as the laboratory values in the EHR on which health care professionals rely and spend the bulk of their time. Although it can be burdensome and distracting for clinical care, the EHR has tremendous untapped potential to support patient-centered care. Realization of this capability is a pressing public health need requiring the full collaboration of technological experts with a broad range of clinical leaders, users, patients, and families to achieve solutions that are both principled and practical. Our new Patient Values Tab is a step forward in this important direction.

## Appendix

Multimedia Appendix 1 Table S1. Mapping the Patient Values Tab content to source documentation.

## Abbreviations

APP: advanced practice providers
EHR: electronic health record
GI: gastrointestinal
HCP: health care proxy
ICU: intensive care unit
MSK: Memorial Sloan Kettering Cancer Center
