# Supplementary material for: A Novel Patient Values Tab for the Electronic Health Record: A User-Centered Design Approach
Source: J Med Internet Res. 2021 Feb 17;23(2):e21615. doi: 10.2196/21615 (PMC7929751; doi:10.2196/21615)
Supplement: Multimedia Appendix 1 [file jmir_v23i2e21615_app1.docx]

**Table S1.** Mapping the Patient Values Tab content to source documentation.

| General category | Subcategory | Data source (general) | Data source (specific) | Display considerations | Comment |
| --- | --- | --- | --- | --- | --- |
| Values summary | Values summary | “Assessment, Patient Personal Values” nursing clinical document | Whole document (including date) | Most recent immediately viewable | Provide link to access the older documents |
| Family and  surrogate decision makers | Decision makers | Care questionnaire^a^  🡪 nursing clinical document | “Who helps you make decisions about your medical care?” | Asked once | Free text |
| Family and  surrogate decision makers | HCP^b^ | Specified patient representative clinical document | HCP name, relationship to patient, and contact information | Most recent | Provide link to access the older documents |
| Family and  surrogate decision makers | HCP | HCP form in advance directive folder in the  EMR^c^ | HCP form scanned into EMR | Most recent | Provide link to access the older documents |
| Family and  surrogate decision makers | Emergency contact | N/A^d^ | Patient info tab in the EHR^e^ 🡪 addresses,  phones,  contacts 🡪 emergency contact | Static text (name, relationship, contact number) | Obtained on registration from Patient Financial Services |
| Family and  surrogate decision makers | Next of kin | N/A | Patient info tab in the EHR 🡪 addresses,  phones,  contacts 🡪 next of kin | Static text (name, relationship, contact number) | Obtained on registration from Patient Financial Services |
| Social history | Living situation | Care questionnaire  🡪 nursing clinical document | “Which one of the following would best describe your current living situation?” | Asked once | Closed-ended items (living alone, living with family or partner, nursing home, etc) |
| Social history | Employment situation | Care questionnaire  🡪 nursing clinical document | “How would you describe your current employment situation?” | Asked once | Closed-ended items (employed, not employed) |
| Social history | Sexual orientation | Care questionnaire  🡪 nursing clinical document | “Do you think of yourself as:” | Asked once | Closed-ended items (straight/heterosexual, gay, lesbian, bisexual, etc) |
| Spiritual and cultural history | Practices and beliefs | Care questionnaire  🡪 nursing clinical document | “Are there any religious, cultural or spiritual practices that are important to you during your course of treatment?” | Asked once | Yes/no |
| Spiritual and cultural history | Practices and beliefs | Care questionnaire  🡪 nursing clinical document | “Do you have any religious, spiritual or cultural beliefs that would affect your care?” | Asked once | Yes/no |
| Spiritual and cultural history | Religious affiliation | N/A | Patient info tab in the EHR 🡪 demographics and visit data 🡪 religion | Static text | Obtained on registration from Patient Financial Services |
| Communication preferences | Preferred name | Care questionnaire  🡪 nursing clinical document | “What name would you like the staff to call you?” | Asked once | Free text |
| Communication preferences | Preferred language | Care questionnaire  🡪 nursing clinical document | “In what language do you prefer to discuss your health care?” | Asked once | Free text |
| Communication preferences | Information preferences | Care questionnaire  🡪 nursing clinical document | “I prefer to receive information:” | Asked once | Closed-ended items (with a lot of detail, as a broad overview) |
| Communication preferences | Information preferences | Care questionnaire  🡪 nursing clinical document | “I prefer to receive information:” | Asked once | Closed-ended items (by myself first, with family or friends present) |
| Illness and treatment understanding | Illness understanding | MSK Engage^f^ | “After talking most recently with my oncologist, I expect the following to happen with my cancer” | Most recent immediately viewable | Free text; provide link to access the older documents |
| Illness and treatment understanding | Treatment understanding | MSK Engage | “After talking most recently with my oncologist, I think the purpose of my cancer treatment is” | Most recent immediately viewable | Free text; provide link to access the older documents |
| Illness and treatment understanding | Treatment understanding | MSK Engage | “After talking most recently with my oncologist, I think my cancer treatment will” | Most recent immediately viewable | Closed-ended items (help me live longer, cure my cancer, help me with symptoms I am having because of my cancer); provide link to access the older documents |
| Goals of care discussion notes | Goals of care discussions | Patient and family discussion notes (clinical documents) | Whole documents (including dates) | Most recent immediately viewable | Can be a stand-alone note or part of a progress note; provide link to access the older documents |
| End-of-life care and resuscitation preferences | Resuscitation preference | DNR^g^ order | “Never entered” | (If none on file) | Static text |
| End-of-life care and resuscitation preferences | Resuscitation preference | DNR order | “Active as of (date)” | (If active) | Static text |
| End-of-life care and resuscitation preferences | Resuscitation preference | DNR order | “Rescinded as of (date)” | (If rescinded) | Static text |
| End-of-life care and resuscitation preferences | Advance directives | Advance directive folder within EMR | All documents scanned into advance directive folder within EMR | All documents scanned into advance directive folder within EMR | N/A |
| Link to consult notes | Supportive care consult notes | Clinical documents | Whole documents  (including dates) | Initial and most recent | Provide hyperlinks |
| Link to consult notes | Ethics consult notes | Clinical documents | Whole documents  (including dates) | All | Provide hyperlinks |
| Link to consult notes | Psychiatry consult notes | Clinical documents | Whole documents  (including dates) | Initial and most recent | Provide hyperlinks |

^a^Care questionnaire: survey given to every new outpatient via MSK Engage (see “e” below); feeds into nursing health assessment (a nursing clinical document).

^b^HCP: health care proxy.

^c^EMR: electronic medical record.

^d^N/A: not applicable.

^e^EHR: electronic health record.

^f^MSK Engage: Memorial Sloan Kettering Cancer Center electronic survey platform, accessed through the digital MSK Patient Portal System.

^g^DNR: Do-Not-Resuscitate.
